# Supplementary material for: Expanding Insights: Harnessing Expansion Microscopy for Super-Resolution Analysis of HIV-1–Cell Interactions
Source: Viruses. 2024 Oct 15;16(10):1610. doi: 10.3390/v16101610 (PMC11512423; doi:10.3390/v16101610)
Supplement: Supplementary file 1 [file viruses-16-01610-s001.zip › Supplementary Figures Revision.pdf]

viruses

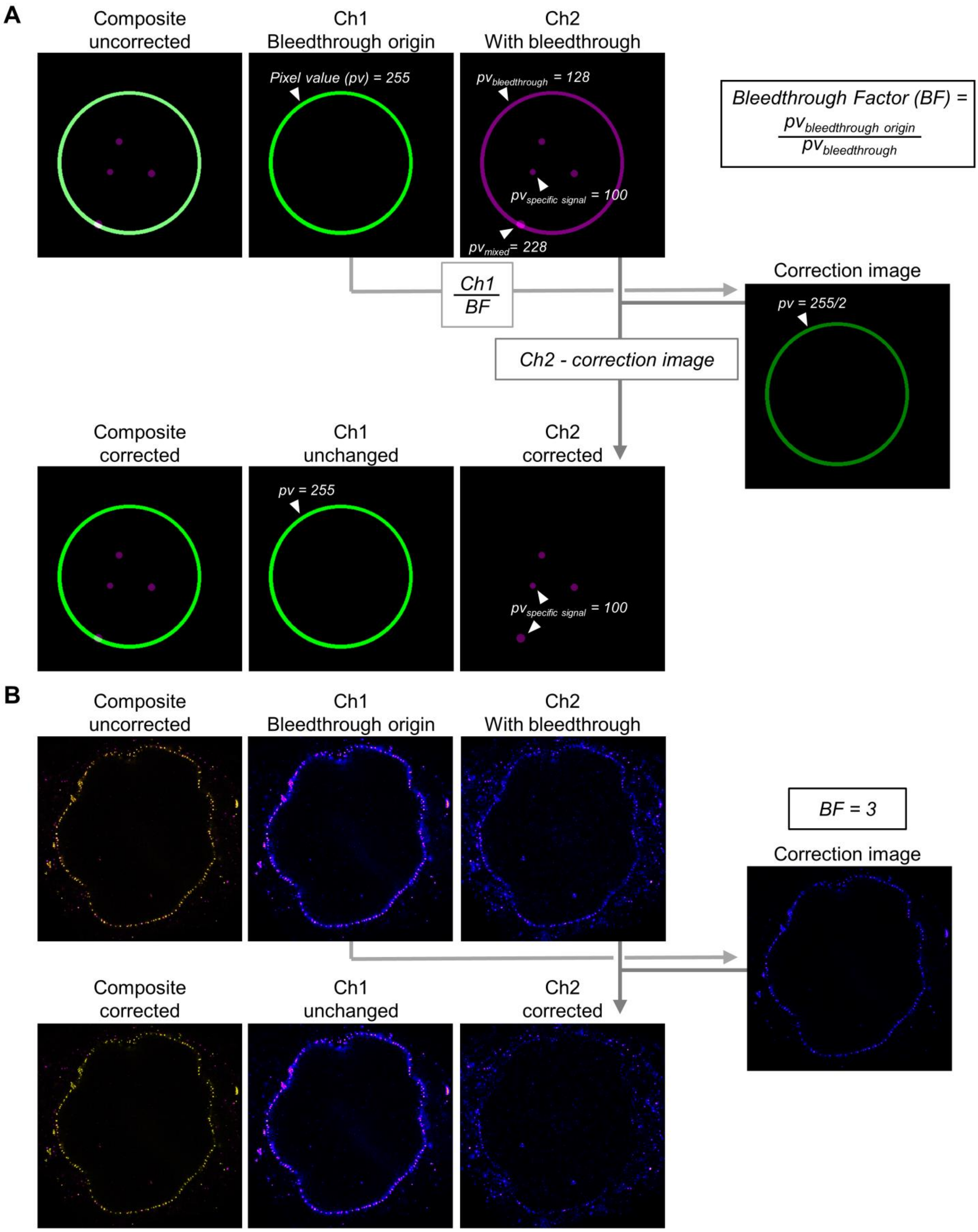
A
Composite
uncorrected
Ch1
Bleedthrough origin
Ch2
With bleedthrough
Pixel value (pv) = 255
pv bleedthrough = 128
pv specific signal = 100
pv mixed = 228
Bleedthrough Factor (BF) = pv bleedthrough origin / pv bleedthrough
Ch1 / BF
Correction image
pv = 255/2
Ch2 - correction image
Composite
corrected
Ch1
unchanged
Ch2
corrected
pv = 255
pv specific signal = 100
B
Composite
uncorrected
Ch1
Bleedthrough origin
Ch2
With bleedthrough
BF = 3
Correction image
Composite
corrected
Ch1
unchanged
Ch2
corrected

Supplementary Figure S1. Overview of bleedthrough correction process. (**a**) Schematic overview of a subtraction-based bleedthrough correction process, where bleedthrough is only present in one channel and a reference channel without bleedthrough is available. (**b**) Example of bleedthrough correction applied to a channel of a TZM-bl cell infected with NNHIV, fixed with PFA/GA at 16 h.p.i., expanded and stained for Nup153 (channel 1, Ch1, yellow) and CA (channel 2, Ch2, magenta). Note that all images are contrast-adjusted to the same range.

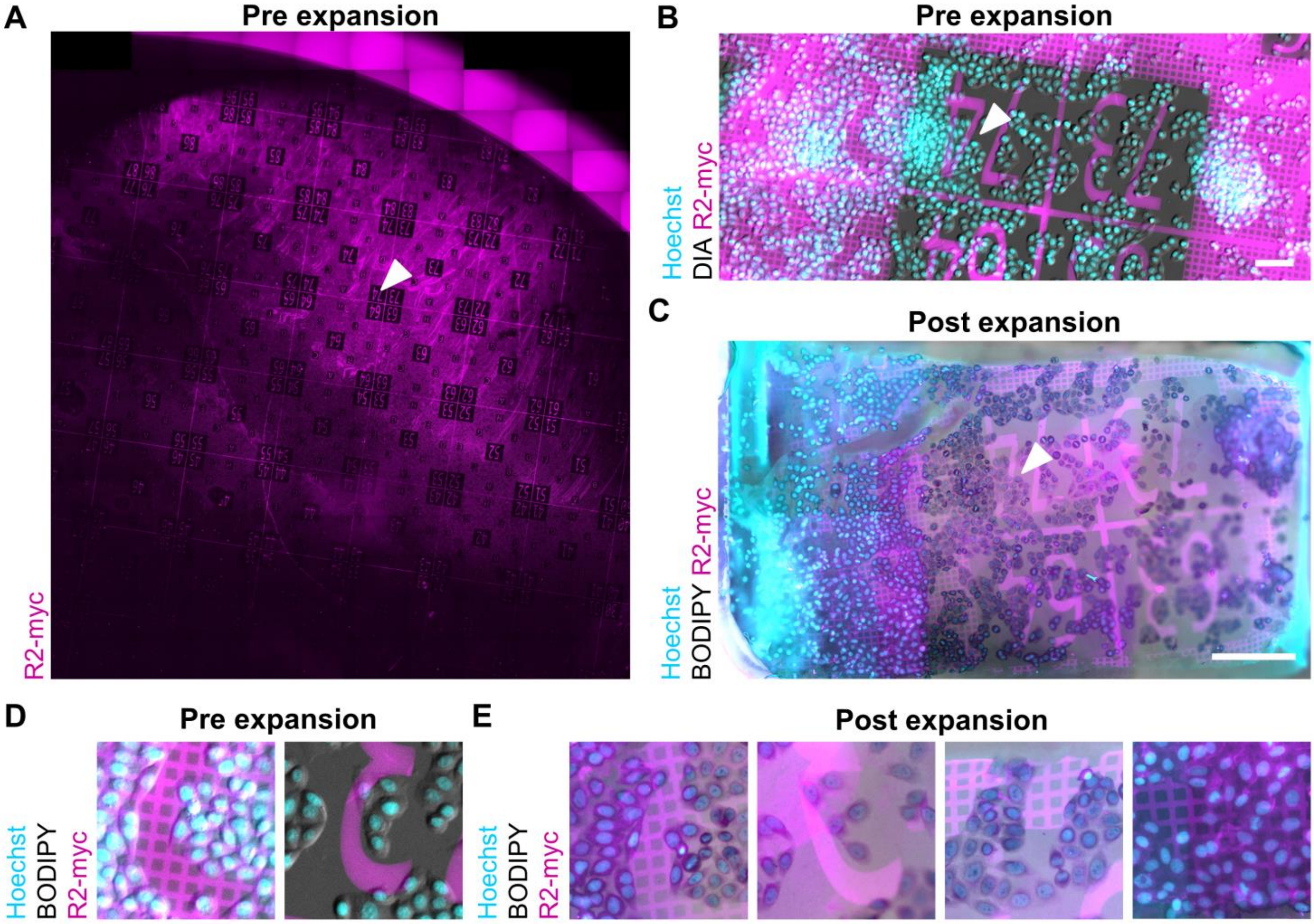

Supplementary Figure S2. Distortion during U-ExM: (**a**) Overview of the RS-myc-his ATTO647N GelMap Grid before expansion. Arrowhead marks the position of the same cluster of cells pre and post-expansion in (**a-c**) (**b**) TZM-bl cells seeded onto an 18 mm GelMap slide patterned with R2-myc-his-ATTO 647N (magenta), fixed with PFA/GA and stained for chromatin (Hoechst, cyan). Scale bar, 100 µm. (**c**) TZM-bl cells seeded onto an 18 mm GelMap slide, fixed with PFA/GA, expanded by U-ExM, and stained for chromatin (Hoechst, cyan), membranes (BODIPY, gray) and R2-myc (magenta). Scale bar, 200 µm. (**d**) Enlarged examples of R2-myc-his-ATTO 647N GelMap grid with fixed TZM-bl cells stained for chromatin (Hoechst, cyan) before expansion. (**e**) Enlarged examples of R2-myc-his-ATTO 647N GelMap grid with fixed TZM-bl cells stained for chromatin (Hoechst, cyan) and membranes (BODIPY, gray) after expansion showing different degrees of distortion.

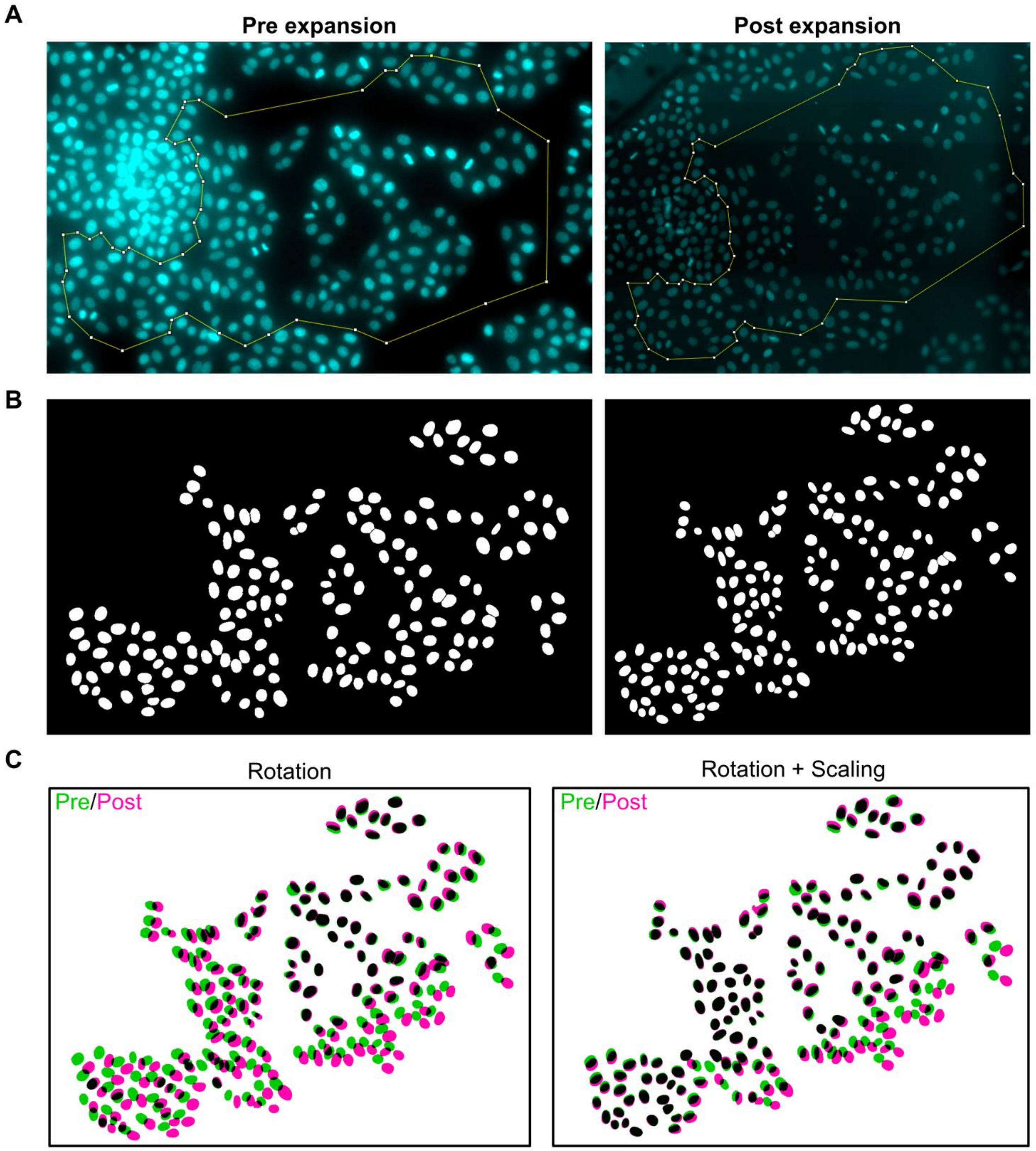

Supplementary Figure S3. Pre and post-expansion measurements of the same set of nuclei: (**a**) TZM-bl cells seeded onto an 18 mm GelMap slide, fixed with PFA/GA, and stained for chromatin (Hoechst, cyan) before (left panel) and after expansion (right panel). Yellow areas mark the same set of nuclei used for measurement of nucleus diameter. (**b**) Binary images of nuclei shown in yellow region in (**a**), segmented using the cellpose 2 *CP* algorithm and eroded by 1 pixel in xy. (**c**). Overlay of the segmented nuclei before (green) and after expansion (magenta), overlaid and aligned either using only rotation (left panel) or rotation and scaling (right panel). Segmentation overlay is shown as black.

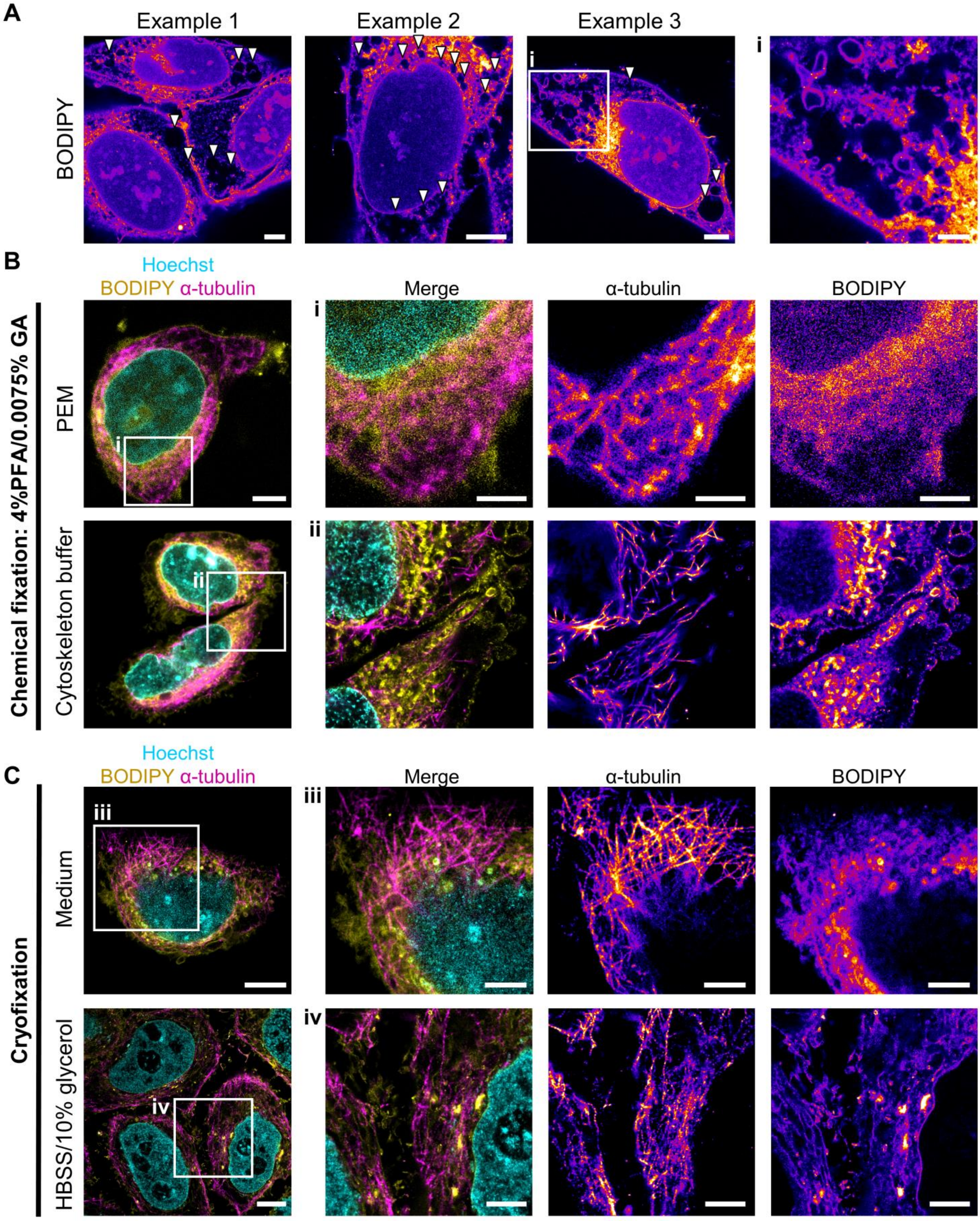

Supplementary Figure S4. Different fixation conditions affect the integrity of the cytoplasm during U-ExM: (**a**) Different examples of disturbed cytoplasmic integrity in U2OS cell fixed with PFA/GA,

expanded and stained for membranes (BODIPY). Arrowheads mark examples of affected areas. Scale bar, 20 µm; scale bar enlargement, 10 µm. (**b-c**) TZM-bl cells fixed with PFA/GA in either PEM or cytoskeleton buffer (**b**) or using cryofixation in either medium or HBSS/10% glycerol (**c**), expanded and stained for chromatin (Hoechst, cyan), membranes (BODIPY, yellow) and α-tubulin (magenta). Scale bar overview, 20 µm; scale bar enlargements, 10 µm.

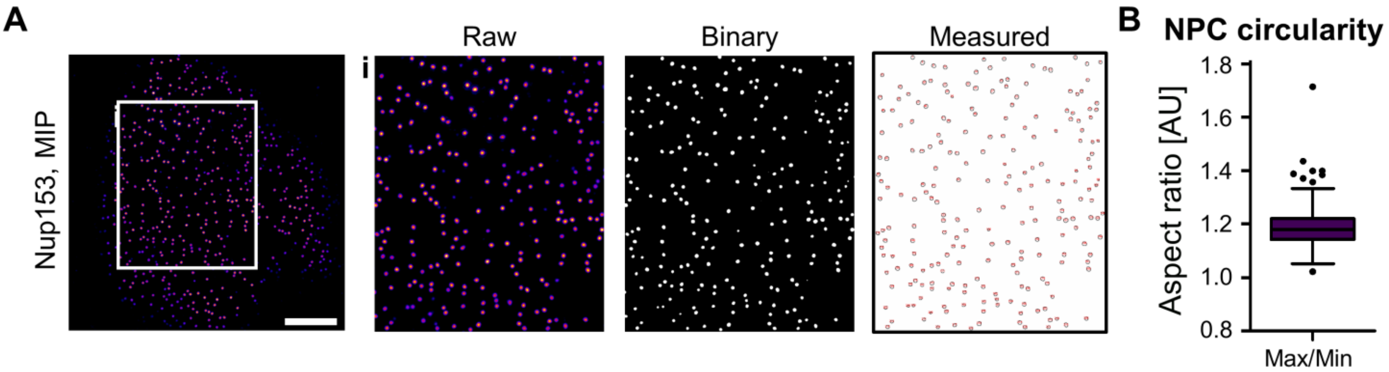

Supplementary Figure S5. Measurement of NPC diameter: (**a**) Different steps in the analysis process to measure the dimensions of the NPC. Overview shows maximum intensity projection (MIP) of a U2OS cells fixed with PFA/GA, expanded and stained for Nup153. Enlargement shows area subjected to image analysis in raw format (left panel), as binary objects (middle panel) and objects included in final measurement (right panel). Scale bar overview; 10 µm. (**b**) Measurement of the NPC aspect ratio as calculated by dividing maximum and minimum Feret diameter of the Nup153 signal as proxy for NPC circularity; n=222; median= 1.18; data blotted according to Tukey method showing outliers as single data points.

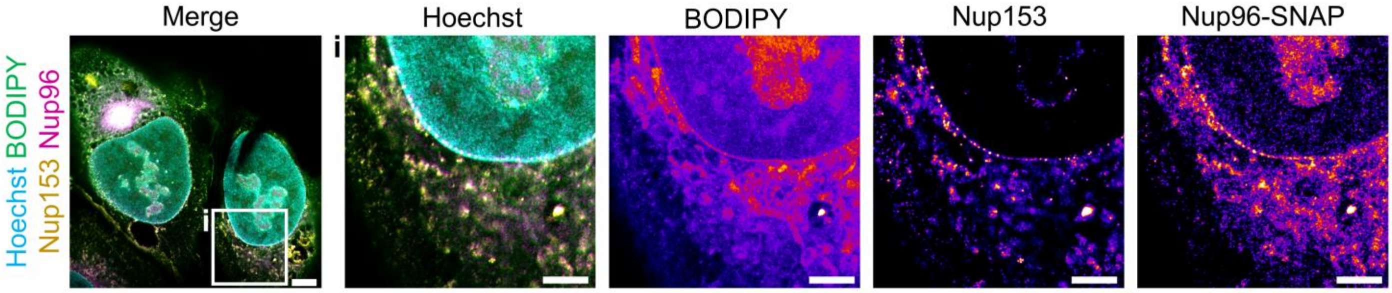

Supplementary Figure S6. SNAP-tagged targets visualized in cells permeabilized with Triton-X100: (**a**) U2OS cells expressing Nup96-SNAP fixed with PFA/GA, permeabilized with Triton-X100 and stained with BG-Biotin before expansion, and staining post-expansion for chromatin (Hoechst, cyan), membranes (BODIPY, green), Nup153 (yellow) and biotin-labeled Nup96 *via* fluorescently conjugated Streptavidin (magenta). Scale bar overview, 20 µm; scale bar enlargement 10 µm.

Additional Supplementary files: 2 videos showing the cells used in Fig. 2B and Fig. 4B.
