# Supplementary material for: Expanding Insights: Harnessing Expansion Microscopy for Super-Resolution Analysis of HIV-1–Cell Interactions
Source: Viruses. 2024 Oct 15;16(10):1610. doi: 10.3390/v16101610 (PMC11512423; doi:10.3390/v16101610)
Supplement: Supplementary file 1 [file viruses-16-01610-s001.zip › Supplementary File S1.pdf]

viruses

MDPI

Ultrastructure Expansion (U-ExM) Microscopy Protocol

The following protocol is based on the protocol provided by Gambarotto et. al. in 2021 [1]. The original protocol also provides several reference images for the construction of a gelation chamber, as well as coverslip handling, which we have omitted here.

Preparations:

1. Stock and working solutions:

- Nuclease-free water (AM9937, Ambion-ThermoFisher)
- Poly-D-Lysine (A3890401, Gibco) – Ready to use (0.1 mg/ml), stored at 4°C
- Ammonium persulfate (APS, 17874, ThermoFisher)
- Tetramethylethylendiamine (TEMED, 17919, ThermoFisher)
- Formaldehyde (FA, 36.5-38%, F8775, SIGMA) – Ready to use – keep at RT
- Acrylamide (AA, 40%, A4058, SIGMA) – Ready to use – keep at 4°C
- N,N′-methylenbisacrylamide (BIS, 2%, M1533, SIGMA). Ready to use – keep at 4°C
- Sodium acrylate (R624-5g, AK Scientific) – keep at -20°C
- Sodium chloride (NaCl, 58.44 g/mol, 11904061, Fischer Scientific)
- TRIS(-BASE) (121.14 g/mol, PUFFERAN® ≥99,9 %, p.a., 4885.2, Roth)
- Sodium dodecyl sulfate (SDS, 288.38 g/mol, Pellets, 20765, Serva)

- Sodium Acrylate Solution: Prepare a stock concentration of 38% (w/v) diluted in ddH_2O (e.g. gradually add 1.9 g of sodium acrylate to 3.1 mL ddH_2O while stirring continuously). Once fully dissolved, aliquot the solution into 500 µL portions and store them at -20°C.

Notes:
While we have successfully used sodium acrylate by AK Scientific, others have also reported good results with other suppliers (SA, 97-99%, 408220 Sigmal Aldrich; 7446-81-3, SantaCruz; A-003-E Sigmal Aldrich).
The 38% (w/v) sodium acrylate solution turns yellow or shows white flocculate when poor purity – do not use sodium acrylate from this batch for expansion and return it. Solutions with the best quality are opaque and colorless.

- 10x Phosphate-buffered saline (PBS) without Ca^{2+} & Mg^{2+} (custom-made: 1.4 M NaCl, 27 mM KCl, 80 mM Na_2HPO_4, 18 mM KH_2PO_4 in ddH_2O), store at RT.

- Monomer solution: For 1 mL of solution, mix 500 µL of sodium acrylate (19% final), 250 µL of AA (10% final), 50 µL BIS (0.1% final), 100 µL 10x PBS. Aliquot (90 µL) and freeze at -20°C– Keep up to 2 weeks. (Prepare Monomer solution latest one day before usage).

Notes:
Note the usage of 10x PBS for the preparation of the Monomer solution.
Please note that the solution will not solidify.

- 5M NaCl stock solution: 292 g NaCl, add 1 L ddH_2O; keep at RT.

- 350 mM SDS stock solution: 10 g SDS, add 80 mL ddH_2O – Caution Work under the fume hood & wear gloves. Add little by little while stirring. Once dissolved, fill up to 100 mL and store at RT.

- Denaturation buffer: 200 mM SDS, 200 mM NaCl, 50 mM Tris in water, pH 9. For a 200 mL solution: 1.2 g of Tris in 10 mL ddH_2O, add 114.28 mL SDS (350 mM stock) and 8 mL NaCl (5 M stock) while stirring. Adjust pH to 9 with HCl and fill up to 200 ml with ddH_2O. Store the buffer at RT.

Notes:
Denaturation buffer can be made well in advance and kept a long time, but the pH has to be adjusted from time to time. It should not have flocculate.

- TEMED and APS solutions (10%): TEMED, mix 100 µL of TEMED to 900 µL of nuclease-free water. APS, dilute 0.1 g into nuclease-free water and fill up to 1 ml. Aliquot (100 µL) and freeze or use fresh – Keep up to 1 month.

Prepare following solutions latest before the ExM day:

- SA stock solution
- Monomer solution
- Denaturation buffer
- TEMED
- APS

2. <u>Coating of imaging slides (if needed for mounting during imaging)</u>

Ibidi glass bottom 8-well slide (80827, ibidi GmbH)
Poly-D-Lysine
ddH_2O

- Pipette 200 µL of Poly-D-Lysine per well of an ibidi 8-well slide.
- Incubate 45 min to 1 h at 37°C.
- Wash 3x with ddH_2O, completely remove any liquid, store in the fridge for ~1 week (longer should also work).

Notes:
Usually done freshly on day of mounting and imaging (leave enough time to dry) or day before imaging.

Expansion protocol:

Day 0:

1. <u>Seeding of cells</u>

- Seed cells on coverslips at the desired density.

Notes:
Although any cell density can be expanded, we recommend using samples with a high confluency (70-90%) as it enhances the ease of identifying cells in expanded samples at high magnification. While coverslips of any size may be used, we suggest opting for 6-10 mm coverslips for the best results, as the gel tends to expand during the process.,

Day 1

Notes:
For planning: The total steps on day 1 take ca. 9.5 h when done optimally.
Until gelation, make sure you know on which side of the coverslip your cells are.

2. Fixing cells

- Wash cells once in 1x PBS or similar buffer solutions.
- Incubate coverslips in fixation solution in a well plate for desired amount of time at desired temperature.
- Wash 3x with 1x PBS, place coverslips into PBS-filled well-plate for storage.
- Prepare all coverslips until this step.

3. Protein crosslinking prevention/anchoring:

- In a well plate, fill wells with 0.7% (v/v) FA/ 1% (v/v) AA in 1x PBS solution, then transfer the coverslips with cells to the wells (1 coverslip per well).
- Fill the surrounding empty wells with water and seal the plate with parafilm to reduce evaporation.
- Incubate for 3.5 h at 37°C.

Notes:
Always prepare the AA/FA solution freshly.
Keep coverslip facing up!
Format/Volume depends on coverslip size (Table 1).
Incubation time can be modified depending on the samples.
AA/FA concentration is also a critical step to play with depending on the sample.
Optional: Perform a regular staining after the crosslinking step to see whether the conditions used did not alter the structure of interest.

4. Gelation:

10% (v/v) TEMED
10% (w/v) APS
Monomer solution
Humid chamber: Dish with a wet paper in the bottom and a parafilm over the wet paper
Ice and metal blocks

- Thaw 10% (v/v) TEMED and 10% (w/v) APS aliquots on ice at least 30 min before gelation.
- Put a humid chamber (with parafilm and wet paper) at -20°C for at least 10-15 min before gelation.
- Take out the plate with the coverslips from the 37°C incubator.
- Place the humid chamber on ice/precooled metal blocks (from now on: stay on ice).
- Take out the coverslips (two in a row, not more), shortly dip coverslips in 1x PBS, remove a little bit of the PBS by leaning the coverslips at an angle to the well plate.
- Add 5 µL of 10% (v/v) TEMED and 5 µL 10% (w/v) APS into the 90 µL monomer solution, vortex 2-3 seconds, make two drops of gelation solution on the parafilm of the humid chamber.

Remarks
Adjust volume of gelation solution for different coverslip size (Table 1)!
It is important to add first TEMED, then APS.

- Rapidly cover each drop with a coverslip with cells facing the gelation solution.
- Incubate 5 min on ice (better gel penetration), cover in aluminum foil from here on.
- Incubate chamber with gels/coverslips at 37°C for 1 h (after this step: all the cells should be included/sticking in the gel).

Notes:
Gel polymerization is very fast! Work very quickly once TEMED and APS are added. Check the remaining solution in the tube to see if gelation occurred.
Prepare everything before adding TEMED and APS
Work at 4°C (on ice)!
Check that parafilm is completely flat in humid chamber; otherwise gel will have thicker and thinner parts and tends to break more easily.
If gelation is done on a thin cover glass slide on the parafilm, the polymerized gels can be imaged to see if cells are present in the gel

5. Denaturation & first round expansion:

Denaturation buffer
1 well per gel
ddH_2O

Note: Format/Volume depends on coverslip size (Table 1).

Denaturation:
- Transfer the coverslips with gels into a well plate filled with denaturation buffer. The orientation of the coverslip is no longer critical at this stage.
- Incubate for 15 min or more at RT with agitation 120 rpm (to allow the gel to detach from the coverslip).
- Preheat heating block to 95°C during this time.
- Transfer the gel into a 1.5 mL reaction tube filled to the top with fresh denaturation buffer (usually ~1.3 ml) (again: the orientation of the cells is not important).

Notes:
This works best with a spoon.

- Incubate for 1.5 h at 95°C.

Notes:
Gels start to expand and becomes wavy during the denaturation – Be gentle when transferring the gel with a spatula/spoon into the 1.5 mL tube. When the gel is strongly sticking to the coverslip, detach it with dull tweezers by sliding it between gel and coverslip carefully(!). You can also shake a bit faster if the gel is not detaching. Often, it takes longer than 15 min for the gel to detach from the coverslip.
Time and denaturation temperature can vary according to the observed organelles (for instance, isolated centrioles require only 30 min at 95°C while mitochondria need to be incubated for 1 h at 70°C [1-2].
Cells are "absorbed" into the gel very close to its surface.
Optional: After denaturation, gels can also be imaged. Any fluorescence present in the initial samples is usually destroyed at this step, but mammalian cells within the gel are still visible in bright-field imaging.

First round of expansion:
- Remove denaturation buffer as much as possible using a 1 mL pipette.

- Transfer the gel in a beaker/dish (or similar container) filled with ample ddH_2O by tilting the tube over the beaker/dish.
- Incubate the gel in water three times for 30 min at RT. To change the water, carefully hold the gel in place with dull tweezers or a spoon while pouring out the water from the beaker/dish. Alternatively, you can use a pipette to remove water.

Notes:
Be gentle with the gel after the first 30 min, it becomes fragile.

Unstained gels can be stored in 1xPBS/0.01% NaN_3 solution at 4°C for few weeks.
Expansion can also be done by incubating 30 min at RT, overnight, 30 min at RT – the key step is to do incubation with water three times for a minimum of 30 min each.

6. <u>Shrinking, blocking and staining of the gels</u>

1x PBS
1x PBS/ 0.5% (v/v) Tween 20 (Roth, 9127.1)
1x PBS/ 3% (w/v) BSA (Albumin fraction V, ≥ 98%, fatty acid free, Roth, 0052.3)
Sodium azide (extra pure, Merck, 1.06688.0250)
Antibodies (Table 3)
Well-plates and dishes

<u>Gel shrinkage for antibody incubation:</u>
- Pour out the water from the beaker/dish while holding back the gel, replace the water with 1x PBS.
- Incubate for 15 min at RT, then exchange with fresh 1x PBS and incubate another 15 min at RT.
- During that time: prepare 1x PBS/ 3%(w/v) BSA solution.
- Measure the diameter of your PBS expanded gel to determine the expansion factor from coverslip to PBS. Measuring can be achieved using a glass plate on top of a laminated piece of millimeter paper.
- On top of a glass plate, cut gel into pieces.
  We prefer 0.5 cm x 1 cm or 0.5 cm x 0.5 cm pieces.

Notes:
Format/Volume depends on coverslip size (Table 1).
We use 0.01% (w/v) NaN_3 in all incubation steps involving BSA or any incubation longer than 30 min.

<u>Blocking:</u>
- Transfer gels into well-plate filled with 1x PBS/ 3% (w/v) BSA.
- Incubate for 30 min at RT with agitation.

Notes:
Handling of gel pieces is best achieved with a spoon and a spatula.
Format/volume of incubation depends on the size of your gel piece (Table 2). It will remain for all your incubation steps. For expansion, transfer the gel into bigger well/dish.

- During that time: prepare primary antibody solution in 1x PBS/ 3% (w/v) BSA (Table 3).
- Replace BSA-solution with primary antibody solution.
- Incubate overnight at RT with agitation.

Day 2

- Wash gel 5x for 10 min with 2x volume 1x PBS/0.5% (v/v) Tween 20 at RT with agitation. The more washing steps to further remove background signal the better.
- During that time: prepare secondary antibody solution in 1x PBS/ 3% (w/v) BSA (Table 4).

Notes:
If nucleic staining is desired, such as with Hoechst dyes, it should be added into the secondary antibody solution (Table 5).

- Incubate the gel in secondary antibody solution for 2.5 h at 37°C with agitation.
- Wash gel 5x for 10 min with 2x volume 1x PBS/0.5% (v/v) Tween 20at RT with agitation.

Notes:
Primary antibody incubation can also be done 2.5-3 h at 37°C, secondary antibody solution can also be incubated overnight at RT.
If you use an antibody for the first time in expansion, use ca. 2x concentrated primary antibody compared to regular immunofluorescence.
Using 1:200 will work for most secondary antibodies.
Antibodies can be spun down at max. speed for 5-10 min at 4°C to achieve better signals.

7. <u>NHS-staining, BODIPY staining and final expansion</u>

- *Optional*: NHS-ester staining - stain at 1:2000 (2 µg/ml) in 1x PBS for 1.5 h at RT under agitation, then wash 3x for 10 min with 2x volume 1x PBS/0.5% (v/v) Tween 20at RT with agitation.

Notes:
NHS-ester staining should be conducted after antibody staining, as performing it beforehand or simultaneously may saturate epitope sites, potentially interfering with antibody binding.

- Second round of expansion: Put the gel in an appropriate container (Table 3) with ddH_2O for 30 min and then exchange it once more for overnight.
- *Optional*: BODIPY staining - stain at 1:500 (2 µM) in ddH_2O overnight during second expansion. Add another 30 min incubation step for washing after staining, e.g. 1x 30 min, overnight with BODIPY, 2x for 30 min on the next day.
- Store at 4°C in ddH_2O/0.01% NaN_3 the next day. Beware, gels are not very stable.

Day 3

8. <u>Mounting and imaging</u>

Poly-D-Lysine coated 8 well ibidi µ-slide

- Determine the dimensions of the gel piece to calculate the expansion factor from PBS to final size to determine the overall expansion factor.
- Check on which side of the gel the cells are by inspecting the gels on an uncoated surface under a fluorescence microscope at low magnification.

Notes:
Optimal results are achieved when mounting occurs just prior to imaging.

We usually inspect both sides of the gel using the lid of an 8-well ibidi slide or a round 35-mm ibidi dish, which is reused for this purpose. BODIPY or Hoechst staining provides the best reference here.
To find the cells within the gel, a standard upright cell culture microscope equipped with LED lighting and appropriate fluorescence filters works well at 10x magnification.

- Cut out a piece of the gel which will fit into one well of an 8-well ibidi slide.
- Place the gel piece with cells facing the Poly-D-Lysine- coated well.
- Gently press/arrange gel so no bubbles are below the gel and the gel surface is attached at all points.
- Add a few drops of water to gels to prevent drying/evaporation.
- Image cells.

Notes:
To easily locate the position of the gel piece in the well, consider marking its location on the lid of the 8 well ibidi slide. This will serve as a helpful reference, as the gels become invisible when submerged in water.
In our hands, especially when using BODIPY, we noted a strong autofluorescence at the bottom of the imaging dish. We generally used this autofluorescence as a reference point during imaging, as cells were generally easiest to find 10-15 µm above this autofluorescent layer. If cells cannot be found during imaging, despite their clear visibility in the cell culture microscope, then it is most often that the gel had detached from the bottom of the imaging dish or the gel was mounted in the wrong orientation.
Stained gels can be stored for ca. 10 days at 4°C in ddH$_2$O/0.01% NaN3.

Additional considerations:

Depending on the size of the coverslip and the size of the gel pieces excised after PBS shrinkage, you will be working with different well plates and incubation volumes. For reference, we work with the following formats and incubation volumes:

Table S1. Container format and incubation volume for different steps of the expansion process.

Step	Coverslip size	Format	Volume
Anchoring	6 mm	48 well plate	0.5 mL
	10-12 mm	12 well plate	1 mL
Gelation solution	6 mm	-	10 µL
	10 mm	-	25 µL
	12 mm	-	35 µL
Denaturation/detachment from coverslip	6 mm	12 well plate	1 mL
	10-12 mm	6-well plate	2 mL
First expansion	6-12 mm	Beaker/10 cm dish	>20 mL
PBS shrinkage	6-12 mm	Beaker/ 10 cm dish	>20 mL

Table S2. Container format and incubation volume for different steps of the staining process.

Step	Gel piece size	Format	Volume
Blocking, antibody and NHS-ester staining	0.5 x 1 cm	24-well plate	250-300 µL
	0.5 x 0.5 cm	48 well plate	200 µL
Washing	0.5 x 1 cm	24-well plate	0.5-1 mL
	0.5 x 0.5 cm	48 well plate	0.5-1 mL
BODIPY incubation/expansion	0.5 x 1 cm	6-well plate	2 mL
	0.5 x 0.5 cm	24-well plate	0.5-1 mL

Antibodies and dyes:

Table S3. Primary antibodies used in this study.

Target	Host Species	Clonality	Source	Cat. Nr.	Dilution
myc-tag	rabbit	polyclonal	Cell signaling technology	2272	1:50
α-tubulin	mouse	monoclonal	Sigma Aldrich	T5168	1:250
SRRM2 (SC35)	mouse	monoclonal	abcam	ab11826	1:250
SON	rabbit	polyclonal	Sigma Aldrich	HPA023535	1:250
Nup153	mouse	monoclonal	abcam	ab24700	1:100-1:250
AHCTF1 (ELYS)	rabbit	polyclonal	Sigma Aldrich	HPA031685	1:50-1:100
Nup88	mouse	monoclonal	BD	611896	1:50
HIV-1 CA1	sheep	polyclonal	[3]	-	1:50
HIV-1 CA1	rabbit	polyclonal	[4]	-	1:50
CPSF6	rabbit	polyclonal	Atlas Antibodies	HPA039973	1:300

Table S4. Secondary antibodies used in this study.

Fluorophore	Host species	Species reactivity	Source	Cat. Nr.	Dilution
Alexa Fluor Plus 405	goat	rabbit	ThermoFisher	A48254	1:200
STAR 460L	goat	mouse	abberior	ST460L-1001-500UG	1:200
	goat	rabbit	abberior	ST460L-1002-500UG	1:200
STAR ORANGE	goat	mouse	abberior	STORANGE-1002-500UG	1:200
	goat	rabbit	abberior	STORANGE-1001-500UG	1:200
STAR RED	goat	mouse	abberior	STRED-1001-500UG	1:200
	goat	rabbit	abberior	STRED-1002-500UG	1:200
Alexa Fluor 647	donkey	sheep	ThermoFischer	A-21448	1:200

Table S5. Dyes, ligands and general stains used in this study.

Reagent	Source	Cat. Nr.	Dilution
Hoechst 33342	ThermoFisher	H1399	1:100 (0.1 mg/mL)
BODIPY-FL Ceramide	ThermoFisher	D3521	1:500 (2 μM)
BODIPY-TR Ceramide	ThermoFisher	D7540	1:500 (2 μM)
Atto594-NHS-Ester	ATTO-TEC	AD 594-31	1:2000 (2 μg/mL)
Streptavidin STAR RED	abberior	STRED-0120-1MG	1:200 (10 μg/mL)
BG-Biotin	New England Biolabs	S9110S	1:200 (50 μM)

References

[1] D. Gambarotto, V. Hamel, and P. Guichard, 'Ultrastructure expansion microscopy (U-ExM)', Methods Cell Biol, vol. 161, pp. 57–81, Jan. 2021, doi: 10.1016/BS.MCB.2020.05.006.

[2] D. Gambarotto et al., 'Imaging cellular ultrastructures using expansion microscopy (U-ExM)', Nat Methods, vol. 16, no. 1, pp. 71–74, Jan. 2019, doi: 10.1038/S41592-018-0238-1.

[3] J. Hanne, V. Zila, M. Heilemann, B. Müller, and H. G. Kräusslich, 'Super-resolved insights into human immunodeficiency virus biology', 2016. doi: 10.1002/1873-3468.12186.

[4] D. A. Bejarano et al., 'HIV-1 nuclear import in macrophages is regulated by CPSF6-capsid interactions at the nuclear pore complex', Elife, vol. 8, 2019, doi: 10.7554/ELIFE.41800.
